# Safety and Efficacy of Remogliflozin in People With Type 2 Diabetes Mellitus: A Systematic Review and Meta-Analysis

**DOI:** 10.7759/cureus.66145

**Published:** 2024-08-04

**Authors:** Jay Tewari, Khalid Ahmad Qidwai, Anadika Rana, Ajoy Tewari, Vineeta Tewari, Anuj Maheshwari

**Affiliations:** 1 Internal Medicine, King George’s Medical University, Lucknow, IND; 2 Internal Medicine, Aasra Hospital, Lucknow, IND; 3 Internal Medicine, King George's Medical University, Lucknow, IND; 4 Internal Medicine, Hind Institute of Medical Sciences, Lucknow, IND; 5 Diabetes and Endocrinology, Jai Clinic and Diabetes Care Center, Lucknow, IND; 6 Anatomy, Era's Lucknow Medical College and Hospital, Lucknow, IND; 7 Medicine, Hind Institute of Medical Sciences, Lucknow, IND; 8 Medicine, Shri Hari Kamal Diabetes and Heart Research Clinic, Lucknow, IND

**Keywords:** meta-analysis, sglt-2i, sglt-2 inhibitor, t2dm, diabetes, remogliflozin

## Abstract

Remogliflozin is a novel SGLT-2 inhibitor used for the management of Type 2 Diabetes Mellitus (T2DM). Since its introduction medical literature is scarce on its quantitative effects. We performed this meta-analysis to ascertain its safety and efficacy in the treatment of T2DM.

Following Preferred Reporting Items for Systematic Reviews and Meta-Analyses (PRISMA) guidelines and the Cochrane Handbook, six studies involving 1,605 participants were analyzed. Our analysis found comparable reductions in glycated hemoglobin (HbA1c) by remogliflozin in comparison to the comparators. It was found to be inferior to other anti-diabetic drugs in decreasing fasting plasma glucose and post-prandial glucose. A significant reduction was obtained in body weight and a significant increase was also found in high-density lipoprotein cholesterol (HDL-C) levels. Remogliflozin did not significantly increase the risk for total adverse events, severe adverse events, or hypoglycemic episodes.

The results were accompanied by high heterogeneity, which necessitates conducting high-quality randomized control trials for more robust evidence synthesis. Overall Remogliflozin can be considered a safe drug with beneficial effects on body weight and HDL-C levels for the treatment of people with T2DM.

## Introduction and background

Remogliflozin inhibits specifically the subtype 2 of the sodium-glucose co-transporter (SGLT2). Initially discovered in 2009, it was approved for the management of type 2 diabetes mellitus (T2DM) in India in 2019. Given in the form of remogliflozin etabonate, which is an ester prodrug of remogliflozin, it enhances the elimination of glucose through urine. It reduces blood glucose levels by blocking SGLT2 channels located in the proximal tubules of the kidneys [1]. It is typically well-received without significant adverse effects [2]. Due to its relatively short half-life (peak concentration achieved in 0.5 to 1 hour), taking it twice daily (100 mg twice a day) is more effective than once-daily dosing [3].

T2DM is a chronic condition caused by either reduced insulin secretion or resistance to its action in peripheral tissue. It can also result because of a combination of these two mechanisms [4]. The prevalence of T2DM is increasing, particularly in South-Asian nations, where India is often identified as having the highest rates worldwide. Globally, 537 million persons aged 20 to 79 were estimated to have been diagnosed with diabetes in the International Diabetes Federation's 2021 report. Estimates indicate that number would rise to 643 million by 2030 and 743 million by 2045 [5]. 

Persistent hyperglycemia due to treatment inadequacy or non-adherence to treatment in uncontrolled diabetes mellitus can lead to numerous complications, both acute and chronic. Acute complications include hypoglycemia, diabetic ketoacidosis, hyperglycemic hyperosmolar state, and hyperglycemic diabetic coma. Chronic microvascular complications encompass nephropathy, neuropathy, and retinopathy, while chronic macrovascular complications include coronary artery disease (CAD), peripheral artery disease (PAD), and cerebrovascular disease [4].

The initial management of T2DM involves adopting healthy lifestyle habits such as maintaining suitable body weight, engaging in regular physical activity for at least 30 minutes most days, limiting sugar and saturated fat intake, and quitting smoking. These measures have demonstrated effectiveness in preventing or delaying the onset of T2DM and in managing blood glucose levels [6]. In addition, several classes of oral hypoglycemic medications are utilized, including sulfonylureas, meglitinides, biguanides (such as metformin), thiazolidinediones, α-glucosidase inhibitors, dipeptidyl peptidase-4 (DPP-4) inhibitors, and SGLT2 inhibitors [7-9] with metformin generally recommended as the initial treatment for T2DM in most guidelines [10].

Considering its recent introduction in India and the limited research, including only one systematic review and meta-analysis by Dutta et al. in 2021 [11], this systematic review and meta-analysis aims to provide updated information in the medical literature.

## Review

The Preferred Reporting Items for Systematic Reviews and Meta-Analyses (PRISMA) criteria were adhered to, and the Cochrane Handbook for Systematic Reviews of Interventions principles were followed in this meta-analysis. The study protocol is registered with the International Prospective Register of Systematic Reviews (PROSPERO) under registration number CRD42024548756.

Study selection criteria

The Population-Intervention-Controls-Outcome (PICO) criteria were used to identify and select studies for this meta-analysis. Our target population (P) were people with T2DM; the intervention (I) of interest was the administration of remogliflozin as a standalone drug or in combination with other anti-diabetic drugs for T2DM management; the control (C) group included people receiving either a placebo or another approved T2DM medication; the outcomes (O) to be evaluated were the changes in HbA1c, fasting plasma glucose (FPG), postprandial glucose (PPG), changes in lipid profile parameters and adverse events.

Only studies with at least 12 weeks of follow-up and adult patients with T2DM were considered. Excluded studies included different forms of diabetes. Studies that met the eligibility criteria had to have a minimum of two treatment groups: one that received remogliflozin (in the approved daily recommended dose of 200 mg or the most approximate dose) (alone or in combination with other oral hypoglycemic agents, OHAs), and the other that received a placebo or another OHA (alone or in conjunction with a normal diabetic treatment regimen). 

Primary and secondary outcomes

The primary outcomes to be evaluated were the changes in HbA1c from baseline after treatment. The secondary outcomes we evaluated were the changes from baseline in fasting plasma glucose (FPG), post-prandial glucose (PPG), body weight (BW), lipid profile, total adverse events (TAE), severe adverse events (SAE), and hypoglycemic episodes (HE) as reported by the authors.

Search strategy

An electronic search was undertaken in Pubmed, Embase, and Cochrane Central databases (from inception until 5th June 2024) using the search terms: (remogliflozin) AND (diabetes). 

Data extraction and study selection

Data extraction was performed independently by two authors (JT and KAQ) using standard extraction forms. Extracted data covered primary and secondary outcomes and study characteristics (author names, year of publication, country of origin, experimental and comparator doses). Any disagreements were resolved by the third author (AR).

Risk of bias assessment

Three authors (JT, KAQ, and AT) independently assessed the risk of bias using the tool in Review Manager (RevMan) Version 5.4.1 (https://login.cochrane.org/). The assessment was done under the domains of Selection Bias, Performance Bias, Detection Bias, Attrition Bias, Reporting Bias, and Additional Bias. Any discrepancies were resolved by the fourth author (VT).

Measures of treatment effect

Results for continuous variables were reported as mean differences (MD), with conversion from SI units as needed and analysis performed using conventional units. Treatment success and other dichotomous outcomes were represented as risk ratios (RR) with 95% confidence intervals (CI). Post-treatment absolute risk differences were used to characterize adverse outcomes. The MDs of primary and secondary outcomes between the remogliflozin and control groups were compared using RStudio (https://www.rstudio.com/).

Heterogeneity assessment

For the primary and secondary outcomes, heterogeneity was first assessed using forest plots. A Chi^2^ test with N-1 degrees of freedom and a statistical significance threshold of 0.05 was then used. Heterogeneity was also evaluated using the I^2^ test, which has the following interpretations: low heterogeneity = <25%; mild heterogeneity = 25% - 50% moderate heterogeneity = 50% - 75%; significant heterogeneity = >75%. The degree and direction of treatment effects, as well as the quality of the evidence supporting heterogeneity, determined the significance of I^2^ values.

Data synthesis

A random-effects model or fixed-effects model was used to pool the data to analyze the primary and secondary outcomes, which were reported as 95% confidence intervals (95% CI). With RStudio, forest plots were created, with the left side of the graph supporting remogliflozin and the right side supporting control. Statistical significance was attained when the p-value was less than 0.05.

Results

**Figure 1:**
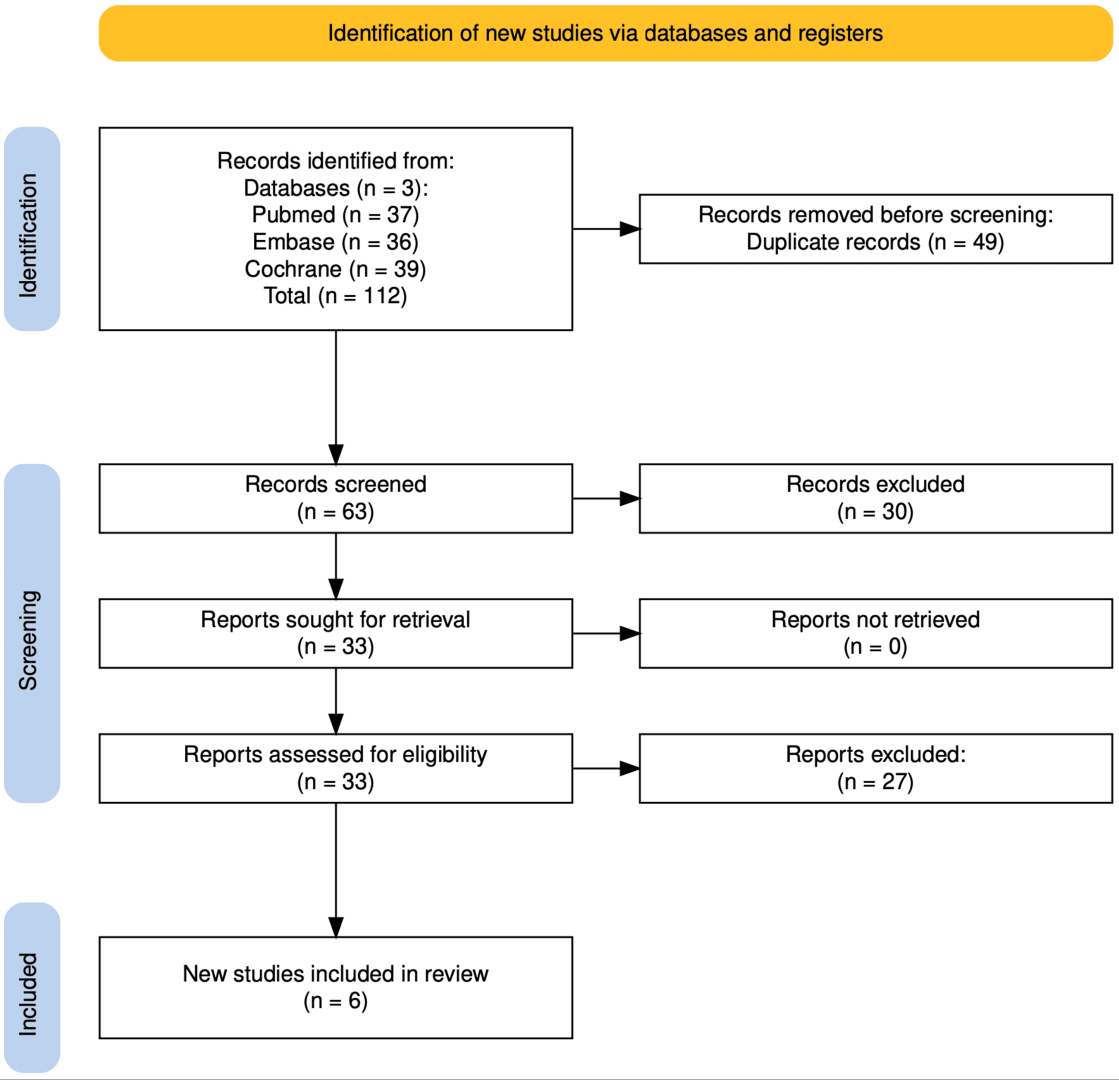
PRISMA study flow chart PRISMA: Preferred Reporting Items for Systematic Reviews and Meta-Analyses

Following our initial search, 112 articles in total were found. Seven articles were selected for inclusion in this meta-analysis after a thorough review based on their complete texts, abstracts, and titles. Six studies ultimately fulfilled all the requirements and were incorporated into the study. Dobbins et al.'s randomized controlled trial (RCT) was excluded since he administered remogliflozin at doses of 500 and 750 mg twice a day [12]. The PRISMA study flow chart has been shown in Figure 1.

The experimental group in the studies by Dharmalingam et al. [13], Sykes et al. (2015a) [14], Sharma et al. [15], Bhosle et al. [16], and Khaladkar et al. [17] was given 100 mg of remogliflozin twice a day. The study groups in the RCT by Sykes (2015) et al. received varied doses of remogliflozin ranging from 100 to 1000 mg daily. We only considered the study group that received 250 mg remogliflozin daily which is close to the recommended dose of 200 mg per day [18].

Studies included in this review used different control groups. Dharmalingam et al. [13], used dapagliflozin 10 mg daily as the control group [13]. Sharma et al. used vildagliptin 50 mg twice daily [15]. Empagliflozin 25 mg with linagliptin 25 mg per day was used as a control in the study by Khaledkar et al. [17]. Sykes et al. (2015) and Sykes et al. (2015a) used pioglitazone 30 mg/day as the comparator [14,18]. Bhosle et al. had four treatment arms, which we designated as Bhosle a; Bhosle b; and Bhosle c; with dapagliflozin 10 mg daily dose, empagliflozin 25 mg once daily, canagliflozin 100 mg doses as the comparator groups respectively [16]. There were no studies that used a placebo as the comparator.

The studies by Dharmalingam et al. and Bhosle et al. had a 24-week follow-up period [13,16]. On the other hand, the follow-up period for the studies conducted by Sykes et al. (2015)[14], Sykes et al. (2015a) [18], and Sharma et al. [15] was 12 weeks. The follow-up period for the Khaladkar et al. trial was 16 weeks [17]. The characteristics of all included studies are shown in Table 1.

**Table 1:** Characteristics of included studies

Author	Country	Year	Study Design	Experimental	Comparator	Follow-up Duration
Dharmalingam et al. [13]	India	2020	Randomized Control Trial	Remogliflozin 200 mg	Dapagliflozin 10 mg	24 weeks
Sykes et al. [14]	United Kingdom	2015	Randomized Control Trial	Remogliflozin 250 mg	Pioglitazone 30 mg	12 weeks
Sykes et al. a [18]	United Kingdom	2015	Randomized Control Trial	Remogliflozin 250 mg	Pioglitazone 30 mg	12 weeks
Sharma et al. [15]	India	2024	Randomized Control Trial	Remogliflozin 200 mg	Vildagliptin 100 mg	12 weeks
Bhosle et al. [16]	India	2022	Comparative Study	Remogliflozin 200 mg	Dapagliflozin 10 mg/ Canagliflozin 100 mg/ Empagliflozin 25 mg	24 weeks
Khaladkar et al. [17]	India	2022	Randomized Control Trial	Remogliflozin 200 mg + Vildagliptin 100mg	Empagliflozin 25mg +Linagliptin 5mg	16 weeks

Risk of bias in the included studies

**Figure 2:**
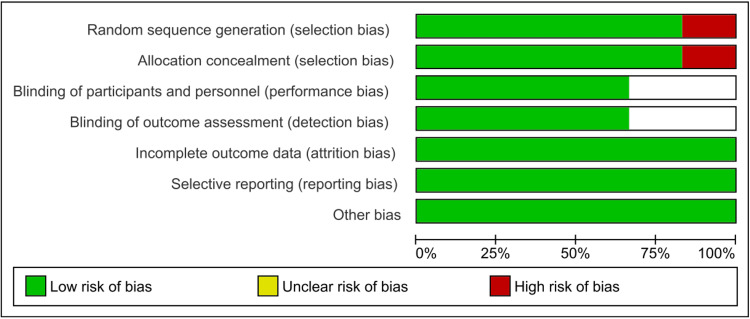
Risk of bias graph

**Figure 3:**
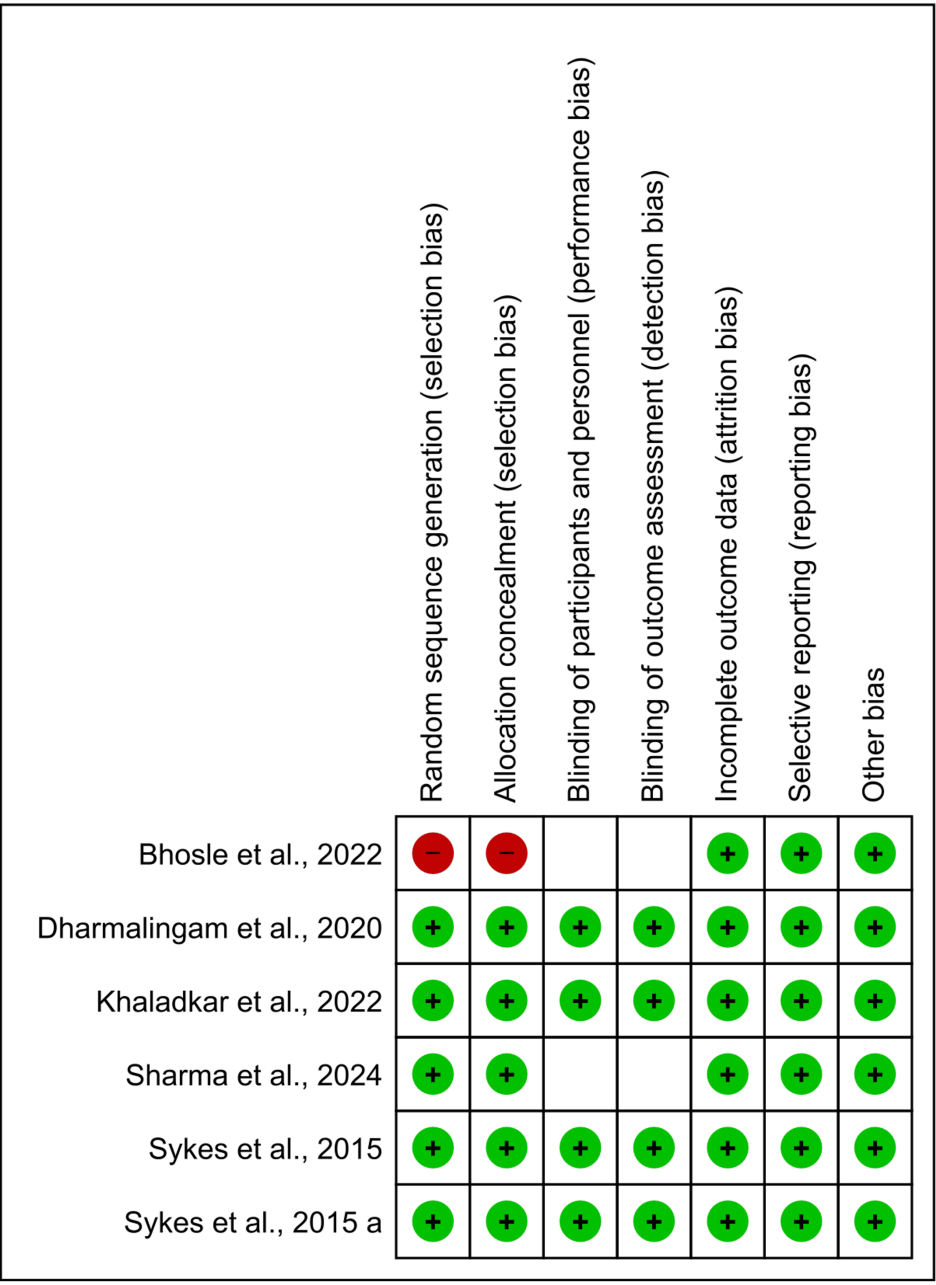
Risk of bias table References: [13-18]

The risk of bias assessment shows that all included studies except the study by Bhosle et al. [16], exhibit low risk across all evaluated domains. The study by Bhosle et al. had a high risk of bias in random sequence generation and allocation concealment, while maintaining a low risk in other areas [16]. Overall, the majority of studies are well-conducted with minimal bias. The summary of the risk of bias assessment done by the RoB v2 tool available in Revman 5.4.1 has been shown in Figures 2, 3.

HbA1c

**Figure 4:**
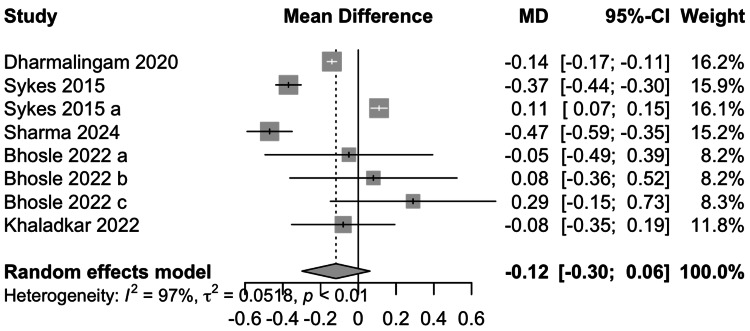
Forest Plot for HbA1c References: [13-18]

Data from six studies involving 1605 participants showed that remogliflozin led to a change of -0.12% HbA1c with a 95% CI of -0.30; 0.06 (Figure 4). Remogliflozin can be considered to cause comparable decreases in HbA1c as compared to the comparators. Although this result was statistically insignificant, significant heterogeneity was present (I^2^=97%). On removing Dharmalingam et al. and Sykes et al. (2015a) studies, I^2^ fell to 77%.

Fasting Glucose (FG)

**Figure 5:**
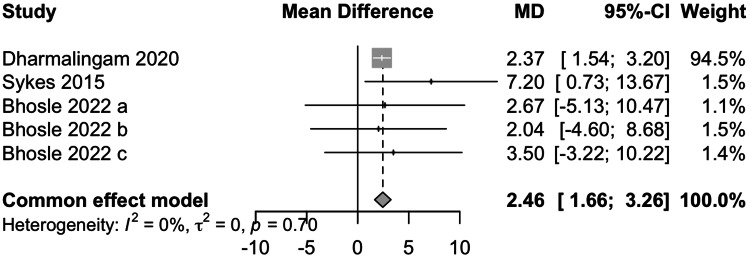
Forest plot for fasting glucose References: [13,14,16]

Data from three studies involving 1057 participants showed that remogliflozin led to a change of 2.46 mg/dL of FG with a 95% CI of 1.66; 3.26 (Figure 5). This result was significant. Low heterogeneity was present (I^2^=0%). Remogliflozin is inferior in decreasing FG as compared to the comparators.

Post Prandial Glucose

**Figure 6:**
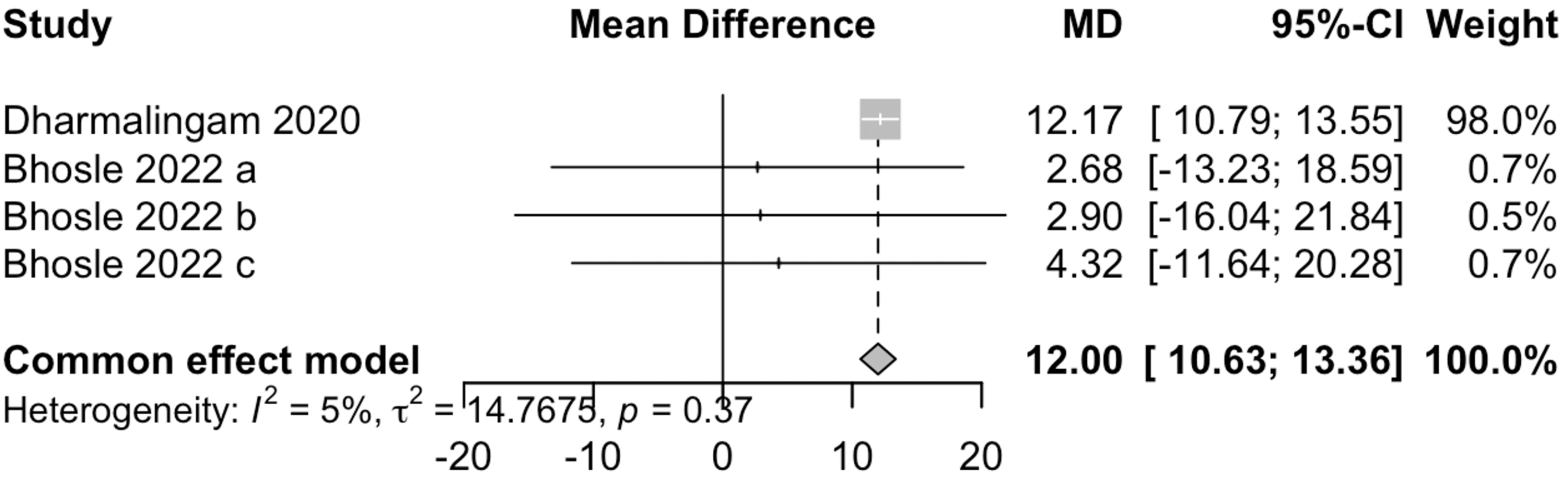
Forest plot for post-prandial glucose References: [13,16]

Data from two studies involving 990 participants showed that remogliflozin led to a change of 12.00 mg/dL of PPG with a 95% CI of 10.63; 13.36 (Figure 6). This result was significant. Low heterogeneity was present (I^2^=5%). Remogliflozin is inferior in decreasing PPG as compared to the comparators.

Body Weight (BW)

**Figure 7:**
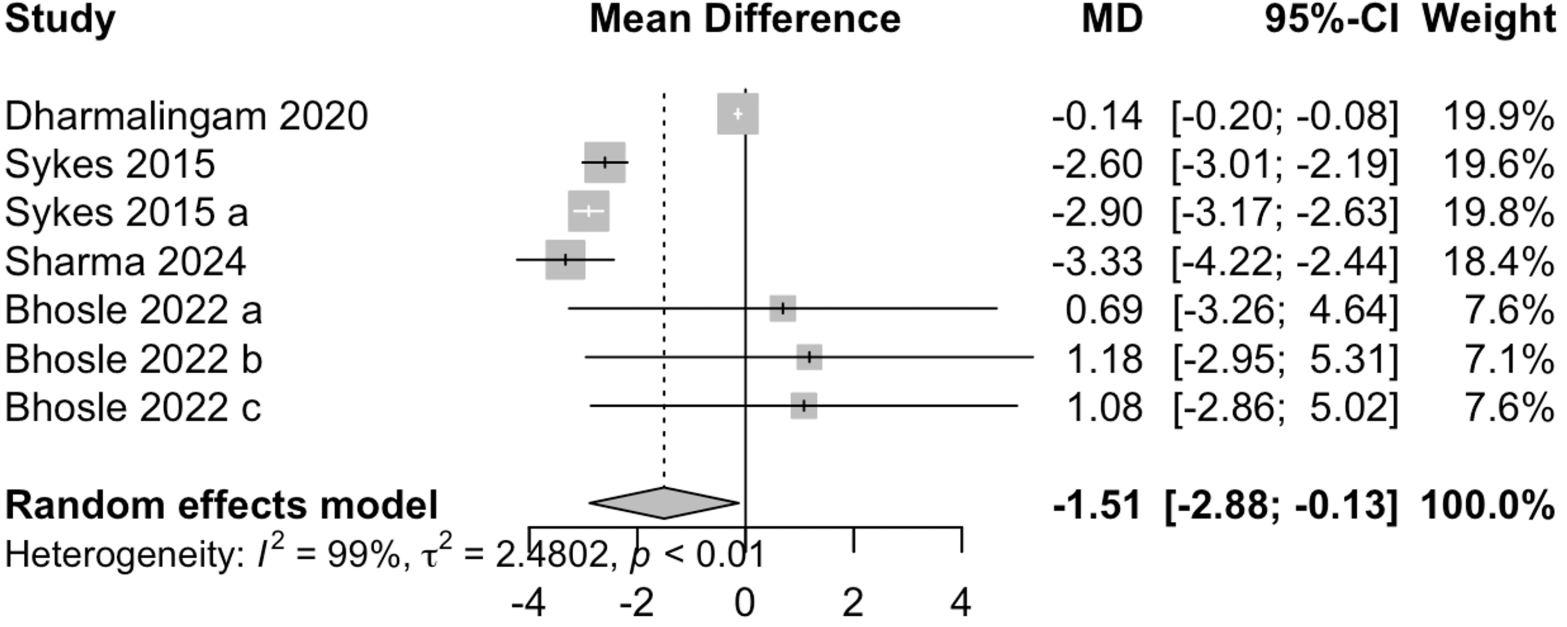
Forest plot for body weight References: [13-16,18]

Data from five studies involving 1205 participants showed that remogliflozin led to a change of -1.51 kg of BW with a 95% CI of -2.88; -0.13 (Figure 7). This result was significant. High heterogeneity was present (I^2^=99%). On removing either of the studies by Sykes et al. and Dharmalingam et al., I^2^ was reduced to 66%. Remogliflozin is superior in decreasing BW as compared to the comparators. 

Total Cholesterol (TC)

**Figure 8:**
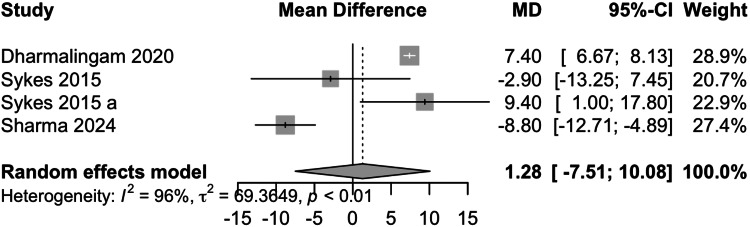
Forest plot for total cholesterol References: [13-15,18]

Data from four studies involving 485 participants showed that remogliflozin led to a change of 1.28 mg/dL of TC with a 95% CI of -7.51; 10.08 (Figure 8). This result was statistically insignificant. High heterogeneity was present (I^2^=96%). On removing the study by Sharma et al., the I^2^ was reduced to 50%. remogliflozin is comparable in decreasing total cholesterol levels as compared to the comparators.

Triglycerides (TG)

**Figure 9:**
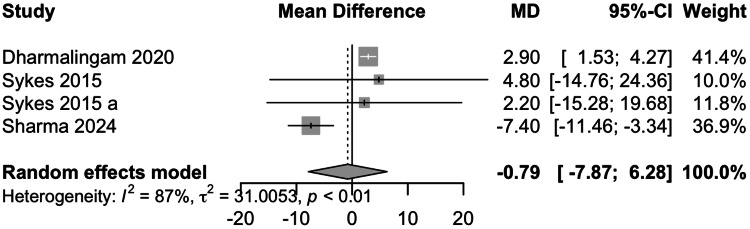
Forest plot for triglycerides References: [13-15,18]

Data from four studies involving 485 participants showed that remogliflozin led to a change of -0.79 mg/dL of TG with a 95% CI of -7.87; 6.28 (Figure 9). This result was statistically insignificant. High heterogeneity was present (I^2^=87%). On removing the study by Sharma et al., the I^2^ was reduced to 0%. Remogliflozin is comparable in decreasing TG levels as compared to the comparators.

High-Density Lipoprotein (HDL) Cholesterol

**Figure 10:**
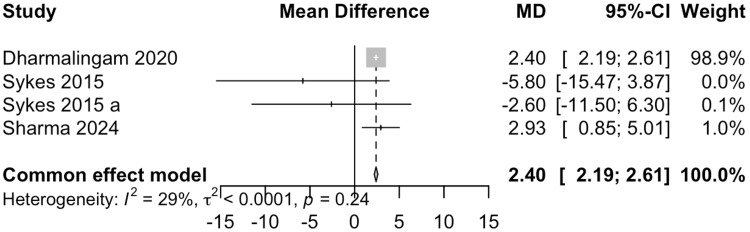
Forest plot for high-density lipoprotein cholesterol References: [13-15,18]

Data from four studies involving 485 participants showed that remogliflozin led to a change of 2.40 mg/dL of HDL cholesterol with a 95% CI of 2.19; 2.61 (Figure 10). This result was statistically significant. Mild heterogeneity was present (I^2^= 29%). Remogliflozin is superior in increasing HDL cholesterol levels as compared to the comparators.

Low-Density Lipoprotein (LDL) Cholesterol

**Figure 11:**
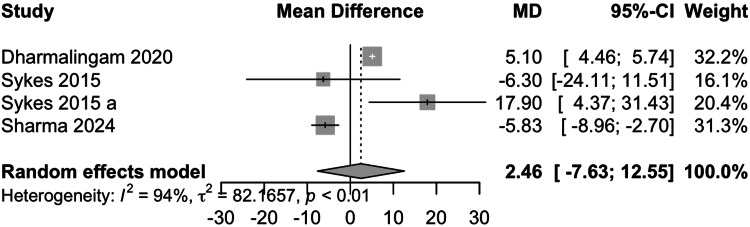
Forest Plot for Low-Density Lipoprotein Cholesterol References: [13-15,18]

Data from four studies involving 485 participants showed that remogliflozin led to a change of 2.46 mg/dL of LDL cholesterol with a 95% CI of -7.63; 12.55 (Figure 11). This result was statistically insignificant. High heterogeneity was present (I^2^= 94%). On removing the study by Sharma et al., the I^2^ was reduced to 60%. Remogliflozin is comparable in decreasing LDL cholesterol levels as compared to the comparators.

Very Low-Density Lipoprotein (VLDL) Cholesterol

**Figure 12:**
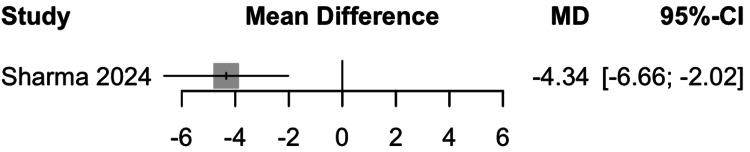
Forest plot for very low-density lipoprotein cholesterol Reference: [15]

Data from one study involving 57 participants showed that remogliflozin led to a change of -4.34 mg/dL of VLDL cholesterol with a 95% CI of -6.66; -2.02 (Figure 12). This result was statistically significant. Remogliflozin is superior in decreasing VLDL cholesterol levels as compared to the comparators.

Total Adverse Events

**Figure 13:**
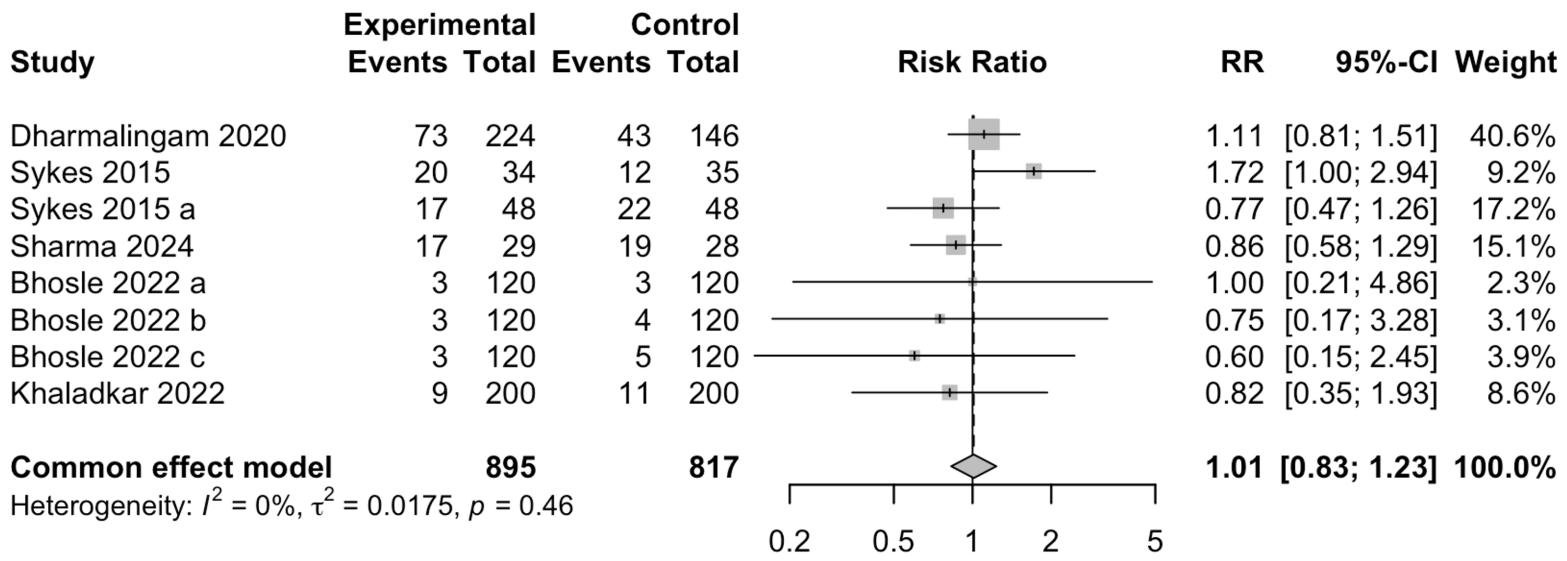
Forest plot for total adverse events References: [13-18]

The data for the occurrence of TAEs was reported in six studies involving 1605 participants. The occurrence of TAEs was not significantly associated with the use of remogliflozin (RR= 1.01; 95% CI: 0.83-1.23) (Figure 13). This result had low heterogeneity (I^2^=0%).

Severe Adverse Events

**Figure 14:**
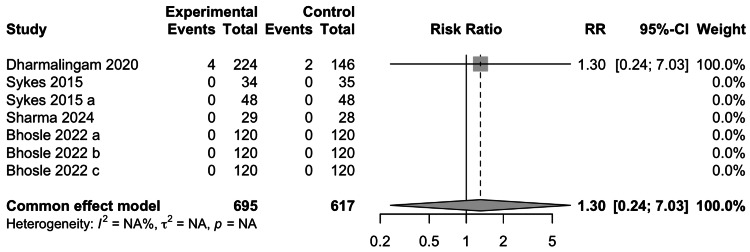
Forest plot for serious adverse events References: [13-16,18]

The data for the occurrence of SAEs was reported in five studies involving 1205 participants. The occurrence of SAEs was not significantly associated with the use of remogliflozin (RR= 1.30; 95% CI: 0.24-7.03) (Figure 14). Of the studies included in the meta-analysis for SAEs, only Dharmalingam et al. found events in both experimental and control groups, whereas in the remaining four studies no events were reported.

Hypoglycemic Episodes

**Figure 15:**
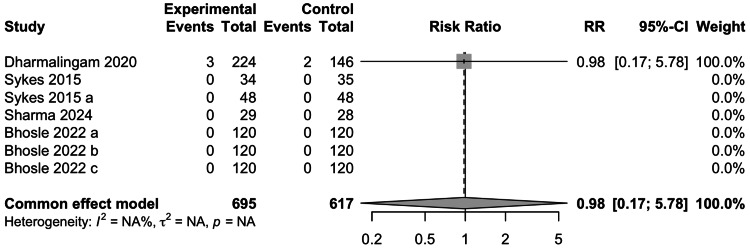
Forest plot for hypoglycemic events References: [13-16,18]

The data for the occurrence of HEs was reported in five studies involving 1205 participants. The occurrence of HEs was not significantly associated with the use of remogliflozin (RR= 0.98; 95% CI: 0.17-5.78) (Figure 15). Of the studies included in the meta-analysis for HEs, only Dharmalingam et al. study found events in both experimental and control groups, whereas in the remaining 4 studies, no events were reported.

Discussion

This meta-analysis assessed the efficacy and safety of remogliflozin in managing T2DM. Remogliflozin was approved exclusively in India in April 2019 [19]. It is a potent and selective SGLT-2 inhibitor that is uniquely administered as a prodrug. Its distinct characteristics include a short half-life, necessitating twice-daily dosing [20]. The American Association of Clinical Endocrinologists and The American College of Endocrinology guidelines recommend SGLT2 inhibitors as one of the first-line treatments for diabetes [21]. Similarly, the American Diabetes Association and the European Association for the Study of Diabetes advise using SGLT2 inhibitors in patients with diabetes who also have co-morbid conditions such as cardiovascular disease and chronic kidney disease [20,22].

Summary of Evidence

Our results indicate that remogliflozin provides similar reductions in HbA1c but is inferior in comparison to other antidiabetic agents in reducing FG and PPG. Remogliflozin is also better at decreasing body weight in comparison to the comparators. Serum HDL levels were also found to increase significantly with remogliflozin use. For VLDL, only one study had reported the mean difference; hence, no meta-analysis could be done, but it showed a significant decrease in VLDL levels as compared to the vildagliptin which was used as a comparator [15]. Our results were met with high heterogeneity for HbA1c, body weight, total cholesterol, TG, and LDL. Although after doing sensitivity analysis the I2 values reduced but were seldom in the low heterogeneity range. All this necessitates the conduction of high-quality RCTs to further delineate remogliflozin’s role in treating people with T2DM.

The meta-analysis by Dutta et al. noted a reduction in body weight with remogliflozin too, but the comparator they had identified was pioglitazone, which has been implicated in weight gain. In our analysis, we found a significant reduction in body weight, but our comparators were not dominated by pioglitazone [11]. 

Our analysis did not find remogliflozin to be associated with any significant risk of adverse events. The number of total adverse events, severe adverse events, and hypoglycemia episodes were comparable between the experimental and control groups. In the systematic review and meta-analysis by Li et al., which evaluated the comparative safety of different sodium-glucose transporter 2 (SGLT2) inhibitors in patients with type 2 diabetes, it was found that remogliflozin and dapagliflozin were associated with a higher incidence of urinary tract infections (UTIs) compared to other SGLT2 inhibitors [23].

For the lipid profile parameters, remogliflozin shows comparable changes with respect to the comparators. However, it leads to a significant increase in HDL cholesterol levels which can be beneficial because of its cardioprotective properties [24]. The meta-analysis by Bechmann et al. and Sánchez-García et al., which evaluated the effect of SGLT 2 inhibitors on lipid profile, shows that SGLT 2 inhibitors increase HDL, LDL, and total cholesterol but decrease the triglycerides level [25,26]. However, their study did not include RCTs of remogliflozin. Our study showed a similar pattern, however, only the meta-analysis of HDL was significant. Four studies included in our study were conducted in India whereas two studies were conducted in the United Kingdom. The effect of SGLT 2 inhibitors on lipid profile slightly varies with ethnicity, Asian ethnicity shows a greater increase in HDL and a greater decrease in triglycerides as compared to Non-Asians [26].

Our meta-analysis builds on the work of Dutta et al. by expanding the number of included studies from three to six and increasing the patient sample size from 428 to 1,605. This expansion also introduces a broader range of comparators. While the three studies analyzed by Dutta et al. included pioglitazone and dapagliflozin, our additional three studies incorporate vildagliptin, canagliflozin, empagliflozin, and a combination of empagliflozin and linagliptin as comparators.

Limitations

This study is limited by significant heterogeneity, a lack of uniformity in comparators, varying strengths of individual studies, and the inclusion of a limited range of ethnicities. 

Implications for clinical practice

As previously mentioned, SGLT-2 inhibitors are utilized as first-line agents for diabetic patients with comorbidities. Based on the findings of our SRMA, physicians might consider incorporating remogliflozin into their treatment plans.

Implications for future research

Consequently, there is a need for high-quality RCTs performed on diverse populations and with uniform comparators. This will result in more robust conclusions regarding the efficacy and safety of remogliflozin.

## Conclusions

This systematic review and meta-analysis comprehensively evaluated the safety and efficacy of remogliflozin in the management of type 2 diabetes mellitus. Remogliflozin was found to cause comparable reductions in HbA1c levels to other antidiabetic agents but was less effective in lowering fasting and postprandial glucose levels. Also, remogliflozin was found to significantly reduce body weight and increase HDL cholesterol levels, which can be beneficial for cardiovascular health.

Remogliflozin was also found to not significantly increase the risk for adverse events as compared to the comparators. These findings suggest that remogliflozin is a safe and effective option for patients with T2DM, particularly in reducing body weight and improving HDL cholesterol levels. However, the high heterogeneity observed in outcomes such as HbA1c, body weight, total cholesterol, triglycerides, and low-density lipoprotein necessitates the need for more high-quality randomized control trials.

Overall, this review highlights the potential of remogliflozin as a viable treatment for T2DM, particularly in terms of its favorable impact on body weight and HDL cholesterol. However, to establish a more definitive role for remogliflozin, further research is needed to address the current limitations and provide more robust evidence on its efficacy and safety.

## Appendices

Search strategy

The search was done on June 5, 2024. Pubmed keywords were (remogliflozin) AND (diabetes) to get 37 results. Cochrane Central keywords were (remogliflozin) AND (diabetes) to get 39 results. Embase (remogliflozin and diabetes).mp. [mp=title, book title, abstract, original title, name of substance word, subject heading word, floating sub-heading word, keyword heading word, organism supplementary concept word, protocol supplementary concept word, rare disease supplementary concept word, unique identifier, synonyms, population supplementary concept word, anatomy supplementary concept word], total 36 results. Total = 112 results.
